# Hyperthermia Enhances Adeno-Associated Virus Vector Transduction Efficiency in Melanoma Cells

**DOI:** 10.3390/cimb45100537

**Published:** 2023-10-23

**Authors:** Alicja Bieńkowska-Tokarczyk, Anna Stelmaszczyk-Emmel, Urszula Demkow, Maciej Małecki

**Affiliations:** 1 Department of Applied Pharmacy, Faculty of Pharmacy, Medical University of Warsaw, 1 Banacha Street, 02-097 Warsaw, Poland; 2 Department of Laboratory Medicine and Clinical Immunology of Developmental Age, Faculty of Medicine, Medical University of Warsaw, 63a Żwirki i Wigury Street, 02-091 Warsaw, Poland; 3 Laboratory of Gene Therapy, Faculty of Pharmacy, Medical University of Warsaw, 1 Banacha Street, 02-097 Warsaw, Poland

**Keywords:** rAAV, hyperthermia, melanoma, gene therapy

## Abstract

Gene therapy perfectly fits in the current needs of medicine for patients with melanoma. One of the major challenges of gene therapy is to increase gene transfer. The role of hyperthermia in the improvement of AAV (adeno-associated virus) transduction efficiency has been indicated. The aim of the present study was to assess the transduction efficacy of melanoma cell lines (A375, G-361, and SK-MEL-1) with the use of the rAAV/DJ mosaic vector under hyperthermia conditions. The analysis of changes in the transduction efficacy and expression of HSPs (heat shock proteins) and receptors for AAV was performed. The transduction was performed at 37 °C and at 43 °C (1 h). Hyperthermia enhanced gene transfer in all the tested cell lines. The most efficient transducing cell line under hyperthermia was A375 (increase by 17%). G361 and SK-MEL-1 cells showed an increase of 7%. The changes in the expression of the AAV receptors and HSPs after hyperthermia were observed. A key role in the improvement of gene transfer may be played by *AAVR*, *HSPB1*, *HSP6*, *DNAJC4*, *HSPD1*, *HSPA8*, *HSPA9*, *HSP90AB1*, and *AHSA1*. This study showed the possibility of the use of hyperthermia as a factor enabling the stimulation of cell transduction with rAAV vectors, thereby providing tools for the improvement in the efficacy of gene therapy based on rAAV.

## 1. Introduction

Melanoma is a malignant neoplasm originating from neuroectodermal cells, which are melanocytes [1]. The skin is the most common location for melanoma (90–96% cases) [1,2]. Other locations characteristic of the occurrence of melanoma include conjunctiva and uvea, mucous membranes of the oral cavity, pharynx, and genital organs, the meninges, and subungual regions [1]. Molecular tests have demonstrated that melanoma is characterized by the presence of mutations in the signaling pathways which regulate proliferation (*BRAF*, *NRAS*, and *NF1*), growth and metabolism (*PTEN* and *KIT*), resistance to apoptosis (*TP53*), length of replication life (*TERT*), cell identity (*ARID2*), and control of the cell cycle (*CDKN2A*) [3,4]. The incidence of melanoma has been growing since 1970. In 2020, 324,635 new cases of skin melanoma and 57,043 deaths were recorded worldwide [5]. Its high growth rate and high metastatic potential make melanoma one of the most difficult neoplasms to treat, and one with the worst prognosis in an advanced stage (3-year survival rate < 10%) [1,6]. In recent years, treatment efficacy in patients with melanoma has been improved by the use of targeted therapies, such as BRAF, CTLA4, and PD1 inhibitors [7]. However, there is a constant need to develop new medicines and new therapeutic combinations for patients who do not respond to systemic therapy or who develop resistance to treatment [8]. Promising results of melanoma treatment have been obtained by combining hyperthermia with chemotherapy or immunotherapy, where hyperthermia has been shown to stimulate anticancer activity of the medicines used [9,10,11,12].

Hyperthermia is a new therapeutic treatment option used together with standard methods of oncology treatment (surgery, chemotherapy, radiotherapy, and immunotherapy) in order to improve treatment efficacy in numerous neoplasms [13,14]. It involves a controlled increase in the patient’s body temperature from 39 °C to 45 °C for a strictly defined time (typically 30–90 min) [13,15]. Depending on the patient’s body area to be exposed to hyperthermia and on the heat distribution technique, local, regional or whole body hyperthermia may be applied [13,16]. These clinical applications have shown that hyperthermia is generally well tolerated by patients [17]. Hyperthermia causes a number of biological changes in the patient’s body: it increases their blood supply, vascular permeability, and membrane fluency (it has been indicated that the heat effect is additionally dependent on the change in the properties of membrane-bound proteins [18]), activates the immune system, blocks the DNA repair pathways, and activates the synthesis of heat shock proteins (HSPs) [15,19,20]. HSPs are a family of chaperones classified on the basis of molecular weight (small HSPs, HSP40, HSP70, HSP90, and large HSPs) [21]. HSP expression increases under conditions unfavorable to cells (in addition to heat changes), e.g., in nutrient deficiency, the presence of toxins, hypoxia, and UV radiation [21,22]. HSPs are important factors participating in the survival, differentiation, and death of cells. HSPs play an important role in the life cycle of many viruses. They participate in the virus entry in the cells, release of genetic material, replication, gene expression, maturation, storing, and releasing of the viruses. Viruses for which HSPs play a supporting function in infection include both DNA viruses (including adenovirus, hepatitis B virus, and herpes simplex virus) and RNA viruses (including coronavirus, hepatitis C virus, influenza, and poliovirus) [23,24,25]. This is a very useful piece of information in the context of gene therapy since it may cause higher efficiency regarding gene insertion into cells during the use of viral vectors. A sufficiently high transfer of genes to the cells is extremely important, since it permits dose reductions of the vectors used, thereby increasing the therapy’s safety [26]. Hyperthermia and HSPs also play a role in the improvement of AAV (adeno-associated virus) transduction efficiency in cells, which has been demonstrated by studies performed in our laboratory [27,28] and studies by other authors [29,30]. In the HSP family, proteins with the highest significance for the improvement in rAAV transduction efficiency in cells include HSP27, HSP40, HSP70, and HSP90. The role played by the FKBP52-HSP90 complex has also been emphasized [27,28,29,30].

AAVs are small, non-enveloped DNA viruses characterized by a high safety profile, an ability to infect dividing and non-dividing cells, stable and long-term transgene expression, and occurrence of serotypes with varied tissue tropism [31]. It is worth emphasizing that rAAV vectors show high stability at a broad range of temperatures and pH, which has been confirmed by other authors, as well as by our own studies [32,33]. The significance of AAV in gene therapy has been demonstrated by the registered products Glybera, Luxturna, and Zolgensma, with AAV1, AAV2, and AAV9 vectors, respectively [34]. Gene therapy is a promising alternative for the treatment of patients with melanoma. A special importance has been attached to the use of oncolytic viruses (e.g., AAV, adenoviruses, herpes simplex viruses, and lentiviruses) [35]. Our and other authors’ research has shown that rAAV is a promising vector for the transduction of melanoma cells [36,37]. Many gene therapy strategies are used to treat melanoma. Multiple gene delivery approaches have been used to achieve anti-angiogenic effects. Of note are soluble VEGFR3-Fc [38], soluble VEGFR1/R2 [39], and plasminogen kringle 1–5 [40]. In the work conducted by Li B. et al. [39], using VEGFR1/R2 in a mouse model, a 2-fold extension of mean survival time was achieved compared to the control. Past research has also attempted to induce an enhanced immune response against tumors using cytokines, such as INF-α [41]. In the study published by Ren C. et al. [41], in a mouse model, a 60% reduction in lung metastasis was achieved. Adeno-associated virus vectors have also been used to deliver antigens to antigen-presenting cells and thereby induce an immune response against tumor cells expressing that antigen, i.e., a tumor vaccine [42,43]. Proapoptotic therapy is also in the spotlight of scientists. In the work of Lee J-H. et al. [37], a gene encoding the proapoptotic protein BIM enclosed in AAV was used. This treatment showed promising results in vitro. The introduction of protocols based on rAAV vectors and hyperthermia in clinics may contribute to the improvement in melanoma treatment efficacy in patients with the worst prognosis.

The aim of the present study was to assess the transduction efficacy of melanoma cell lines with the use of the rAAV/DJ vector under hyperthermia conditions, and the analysis of correlations between changes in the efficacy of gene transfer and expression of genes encoding HSPs and receptors for AAV. The rAAV/DJ vector is a mosaic vector that was created via shuffling eight different wild-type serotypes (AAV2, 4, 8, and 9, avian AAV, bovine AAV, and caprine AAV). We used this vector due to its high transduction efficacy, regardless of the cell’s origin exceeding the capabilities of the wild-type serotypes [44]. We decided to choose one vector, which will transduce different cell lines. We used cell lines from the melanoma panel designed by the ATCC to evaluate how representative melanoma cell lines respond to the applied hyperthermia conditions. These cell lines have the same tumor origin, but differ in their etiology, morphology, and genomic mutation [45].

## 2. Materials and Methods

### 2.1. Cell Lines

Cell lines of melanoma A375 (CRL-1619), G-361 (CRL-1424), and SK-MEL-1 (HTB-67) from the Melanoma Cancer Cell Panel (TCP-1013) and the cell line of normal fibroblasts CCD-1064Sk were obtained from the ATCC (American Type Culture Collection, Manassas, VA, USA). The A375, G361, SK-MEL-1, and CCD-1064Sk cells were cultured accordingly in Dulbecco’s Modified Eagle Medium (DMEM; Gibco, Thermo Fisher Scientific, Waltham, MA, USA), McCoy’s 5A Modified Medium (Sigma-Aldrich, St. Louis, MO, USA), Eagle’s Minimum Essential Medium (EMEM, Gibco, Thermo Fisher Scientific, Waltham, MA, USA), and Iscove’s Modified Dulbecco’s Medium (IMDM, Gibco, Thermo Fisher Scientific, Waltham, MA, USA). All the media were supplemented with 10% fetal bovine serum (FBS; Gibco, Thermo Fisher Scientific, Waltham, MA, USA) and 1% antibiotic/antimycotic solution (Gibco, Thermo Fisher Scientific, Waltham, MA, USA). The cells were incubated under standard culture conditions (37 °C, 5% CO_2_, and relative humidity 95%).

### 2.2. Transduction of Cells at Hyperthermia

For cell transduction under hyperthermia conditions, the rAAV/DJ/CAG/GFP mosaic vector (rAAV/DJ; Cat. No. 7078; Vector Biolabs, Malvern, PA, USA) was used at the multiplicity of infection (MOI) of 4 × 10^4^ gene copies. This vector encoded eGFP (enhanced green fluorescent protein; GFP) under the control of the CAG promotor (cmv/β-actin). Before starting transduction, rAAV/DJ test tubes were transferred from −80 °C to 4 °C, and then to room temperature.

**Figure 1:**
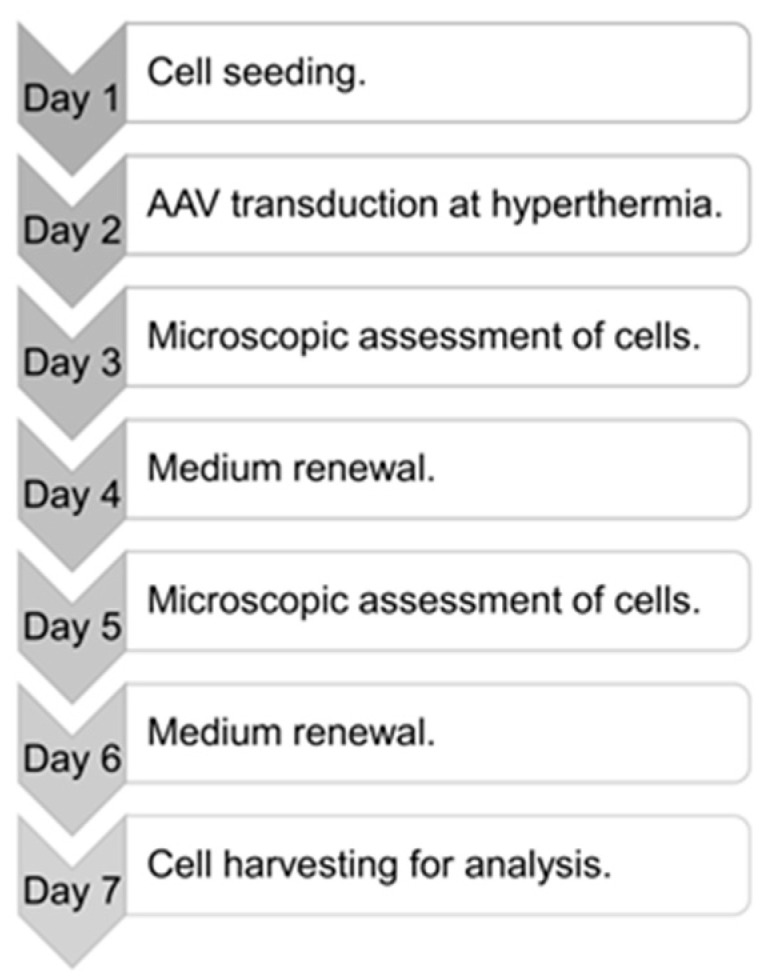
Methodology of melanoma cell transduction with rAAV/DJ vectors under hyperthermia. This experiment lasted seven days; the activities performed on each day are presented in the timeline.

The cells were seeded onto 6 cm plates (Nunc, Thermo Fisher Scientific, Waltham, MA, USA) in the amount of 1.5 × 10^5^ for A-375 and G-361, and in the amounts of 1.0 × 10^5^ and 3.0 × 10^5^ for CCD-1064Sk and SK-MEL-1, respectively. After 24 h of incubation under standard culture conditions, the culture media with 10% FBS were replaced with media with 2% FBS heated to 37 °C and 43 °C, and a previously thawed rAAV/DJ vector was immediately introduced in the medium over the cells. The cells were incubated for 1 h at 43 °C in an incubator (5% CO_2_ and relative humidity 95%). Then, the cells were transferred to standard culture conditions. Control samples (non-transduced and non-exposed to hyperthermia) were performed at the same time. Culture media with 2% FBS were replaced every 48 h. On day 7 after cell seeding, transduction efficiency was assessed. The course of the experiment is shown in Figure 1. The experiment was performed two times in three replicates each time; for every replicate, the evaluation of transduction efficiency was conducted.

### 2.3. Evaluation of Transduction Efficiency

#### 2.3.1. Microscopic Evaluation

In order to evaluate GFP expression, the cells were photographed with the use of an inverted fluorescence microscope (Olympus IX53, Olympus, Tokyo, Japan) with the lighting system pE-300white (CoolLED, Andover, MA, USA). Control and transduced cells were photographed at 10× magnification in FITC (fluorescein isothiocyanate) and BF (bright field) filters. The pictures were taken and edited with the use of cellSens Dimension 1.18 software (Olympus, Tokyo, Japan).

#### 2.3.2. Automatic Cell Counter

The photographed cells were harvested from culture vessels with the use of trypsin (Gibco, Thermo Fisher Scientific, Waltham, MA, USA) and suspended in 1 mL of complete medium. Following this step, 10 μL of cell suspension was collected onto a counting slide (EVE™ Cell Counting Slides, NanoEnTek, Seoul, Korea), which was placed in a Countess II FL Automated Cell Counter (Invitrogen, Thermo Fisher Scientific, Waltham, MA, USA) with an EVOS Light Cube for GFP (470/22 nm excitation; 510/42 nm emission). The device specified the percentage of cells showing GFP fluorescence (GFP+). The test was applied to both transduced and control cells.

#### 2.3.3. Flow Cytometry (FACS)

The evaluation of transduction efficiency in the population of live cells was performed with the use of flow cytometry. For this purpose, the cells harvested from culture vessels were counted in the automatic cell counter and rinsed twice with PBS (Gibco, Thermo Fisher Scientific, Waltham, MA, USA). The cells were suspended in 500 μL of PBS (1 × 10^6^ cells/mL) with the addition of 0.5 μL of 7-AAD dye (7-aminoactinomycin D, Invitrogen, Thermo Fisher Scientific, Waltham, MA, USA), enabling the exclusion of dead cells during the analysis. The test was performed with the use of the flow cytometer BD FACSCanto (Becton Dickinson, Franklin Lakes, NJ, USA) and analyzed with the use of FlowJo™ 10 Software (Becton Dickinson, Franklin Lakes, NJ, USA).

#### 2.3.4. Quantitative PCR (qPCR)

In order to evaluate the number of genome copies of rAAV vectors which transduced cells, a qPCR reaction was conducted. Total DNA was isolated with the High Pure Viral Nucleic Acid Kit (Roche Life Science, Mannheim, Germany), in accordance with the manufacturer’s protocol. DNA was evaluated qualitatively and quantitatively with the use of the Q5000 UV–Vis spectrophotometer (Quawell, San. Jose, CA, USA). qPCR was conducted in the StepOnePlus Real Time PCR System (Applied Biosystems, Thermo Fisher Scientific, Waltham, MA, USA) with the use of a previously developed and validated method based on the use of a probe and primers for the *itr* (inverted terminal repeat) sequence. Reverse primer: 5′-CGGCCTCAGTGAGCGA-3′, forward primer: 5′-GGAACCCCTAGTGATGGAGTT-3′, and probe: 5′-6-FAM-CACTCCCTCTCTGCGCGC-TAMRA-3′ [27,28,32,36,46]. The Taqman^®^ Universal Master Mix II buffer with UNG (Applied Biosystems, Thermo Fisher Scientific, Waltham, MA, USA) was used. The total qPCR volume was 10 μL. The reaction contained 50 ng of DNA and was performed under the following conditions: 50 °C for 2 min, 95 °C for 10 min, 40 cycles at 95 °C for 15 s, and 60 °C for 60 s. In order to determine the number of transcripts, an absolute quantitative analysis was performed with the use of StepOne Software v2.3 (Thermo Fisher Scientific, Waltham, MA, USA). Data were determined quantitatively with the use of a standard curve. In brief, standard curves were prepared via serial dilutions of plasmid pAAV-IRES-hrGFP (Agilent Technologies, Santa Clara, CA, USA), which were used for the calculation of the number of copies of *itr* sequences in the test samples. The standard curve covered the range from 10 molecules to 10^10^ molecules. The number of copies of *itr* sequences was normalized as the number of virus copies per 50 ng of total genomic DNA.

### 2.4. Evaluation of Gene Expression

To evaluate the expression of genes encoding receptors for AAV and HSPs, total RNA was isolated from cells with the use of the High Pure RNA Isolation Kit (Roche Life Science, Mannheim, Germany), in accordance with the manufacturer’s protocol. The qualitative and quantitative evaluations of the isolated RNA were performed with the use of a spectrophotometer (Q5000 UV-VIS, Quawell, San. Jose, CA, USA). The High-Capacity cDNA Reverse Transcription Kit with RNase Inhibitor (Applied Biosystems, Thermo Fisher Scientific, Waltham, MA, USA) was used for the synthesis of complementary DNA; the manufacturer’s protocol was followed. The expression of genes encoding receptors for AAV was examined with the following TaqMan assays (Thermo Fisher Scientific, Waltham, MA, USA): AAVR (Hs00967343_m1), HSPG1 (Hs0181432_m1), and HSPG2 (Hs01078536_m1). β-actin (Hs01060665_g1) was used as an endogenous control. The expression levels of AAV receptors were determined in three biological repeats; each repeat was assayed two times. The expression of genes encoding HSPs was measured with the use of the TaqMan Array 96-Well FAST Plate Human Heat Shock Proteins (Cat. No. 4418733; Applied Biosystems, Thermo Fisher Scientific, Waltham, MA, USA), in which GAPDH (Hs99999905_m1) was used as an endogenous control. The expression levels of *HSPs* were determined for two biological repeats. All qPCRs were conducted in the StepOnePlus™ Real-Time PCR System, in accordance with the manufacturer’s protocol. The analysis of gene expression with ddCt was performed using Expression Suite Software v 1.0.4 (Thermo Fisher Scientific, Waltham, MA, USA).

### 2.5. Statistical Analysis

The results were presented as mean and SD (standard deviation). The results of HSP gene expression after hyperthermia were presented in the form of HSP and SE (standard error). Statistical analysis was performed with the use of GraphPad Prism 7 (GraphPad Software, La Jolla, CA, USA). The results were analyzed with the use of the one-way analysis of variance (ANOVA, α = 0.05) statistical test with the post-hoc Tukey test (α = 0.05) for multiple comparisons. Differences between results were considered significant when *p* < 0.05. Statistical significance was designated with asterisks (* *p* < 0.05; ** *p* < 0.01; *** *p* < 0.001, and **** *p* < 0.0001).

## 3. Results

### 3.1. Melanoma Cell Transduction with the rAAV Vector under Hyperthermia

**Figure 2:**
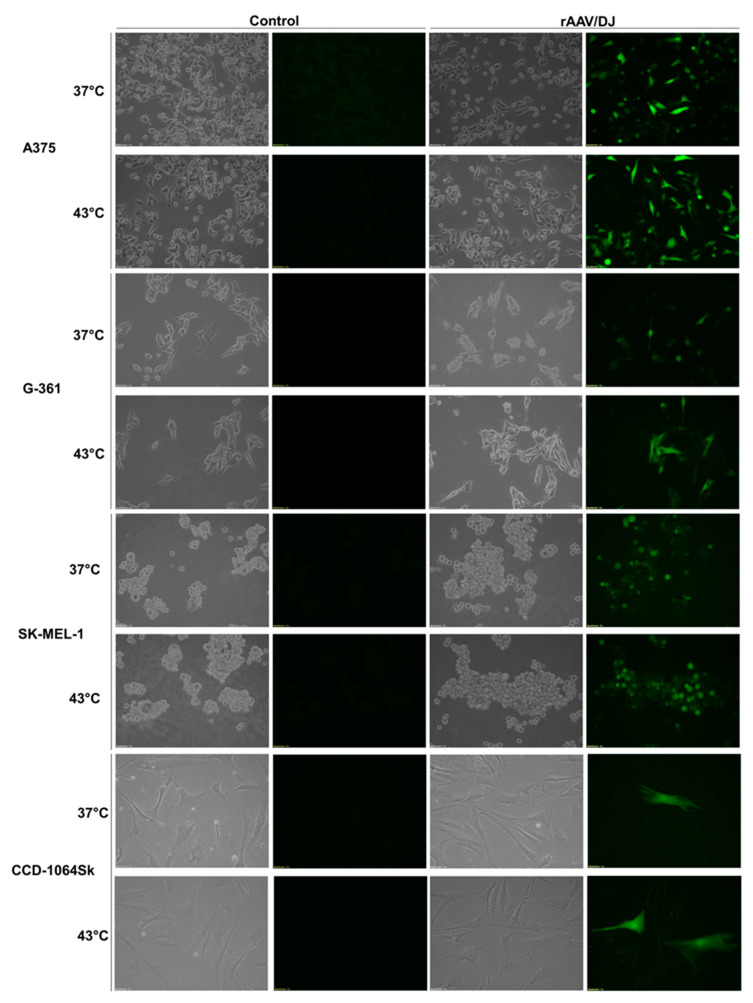
Photographs of the A375, G-361, SK-MEL-1, and CCD-1064Sk cells. These photos show non-transduced cells (control) and transduced cells (rAAV/DJ) under standard conditions (37 °C) and at hyperthermia (43 °C). The photos were taken using a fluorescent microscope in the bright field (BF) and FITC (fluorescein isothiocyanate) filters at a 10× magnification.

**Figure 3:**
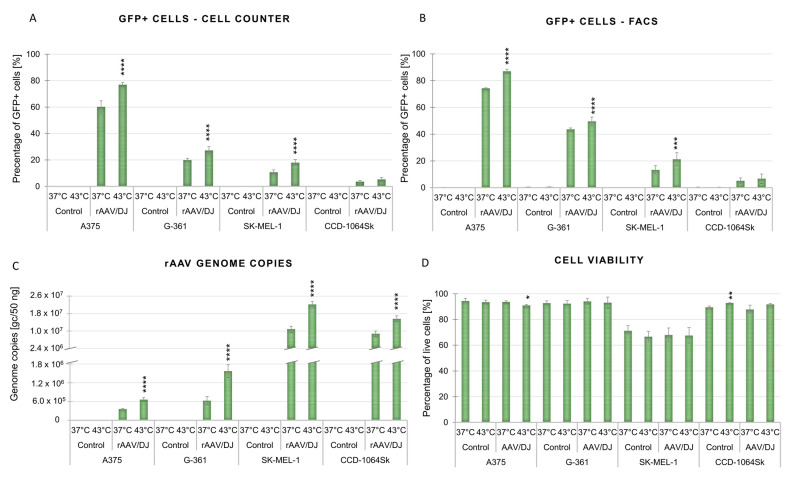
Efficiency of transduction with the rAAV/DJ vector of the A375, G-361, SK-MEL-1, and CCD-1064Sk cells under hyperthermia. The results are shown for non-transduced cells (control) and transduced cells (rAAV/DJ) under standard conditions (37 °C) and underhyperthermia (43 °C). The results are presented as a mean of two independent experiments conducted in three repeats ± SD. The number of GFP+ (green fluorescent protein positive) cells was measured with an automatic cell counter (**A**) and a flow cytometer (FACS) (**B**). (**C**) The number of rAAV/DJ vector genome copies was measured in the analyzed cells with the qPCR method. Statistically significant differences between 37 °C and 43 °C are marked with asterisks (*** *p* < 0.001; **** *p* < 0.0001). (**D**) Cell viability after exposure to the hyperthermia condition. The results are presented as a percentage of live cells. The data were measured with FACS. Statistically significant differences between the number of live cells at 37 °C (control) and values in other conditions are marked with asterisks (* *p* < 0.05; ** *p* < 0.01).

**Figure 4:**
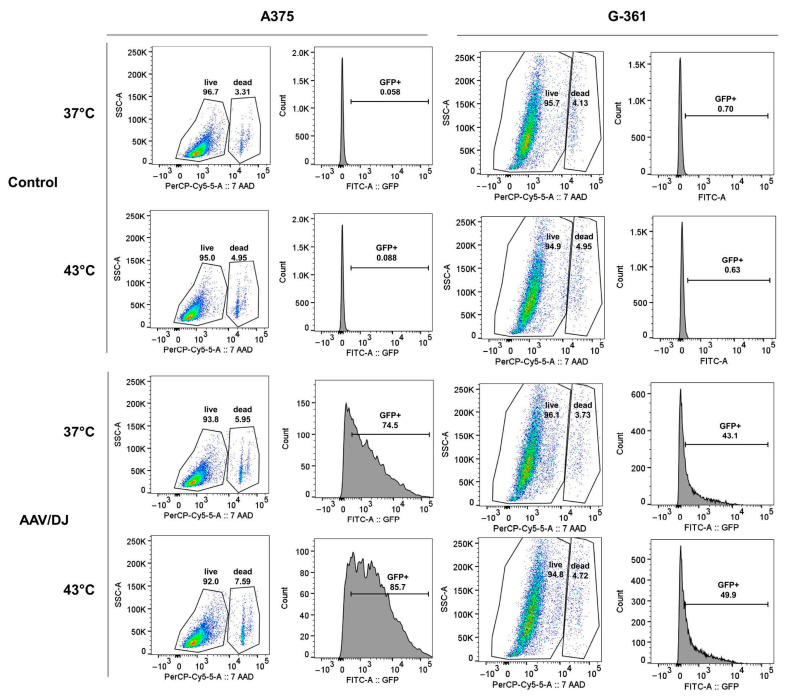
Efficiency of transduction with the rAAV/DJ vector of the melanoma A375 and G-361 cells under hyperthermia. Representative dot plots and histograms present the number of live and dead cells (%) and the number of GFP+ cells (%) for non-transduced cells (control) and transduced cells (rAAV/DJ) under standard conditions (37 °C) and under hyperthermia (43 °C). The percentage of GFP+ cells was measured in the population of live cells.

**Figure 5:**
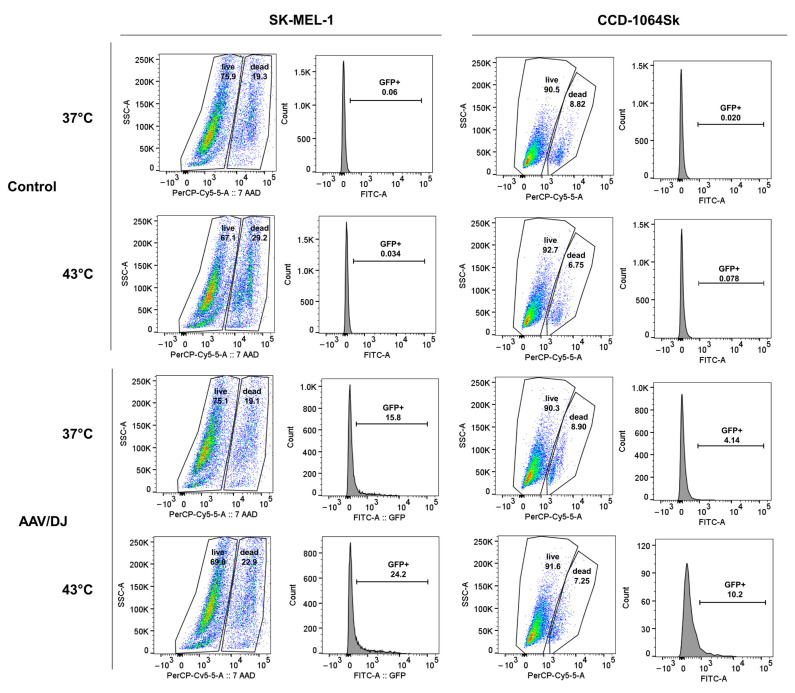
Efficiency of transduction with the rAAV/DJ vector of the SK-MEL-1 melanoma cells and normal fibroblasts CCD-1064Sk under hyperthermia. Representative dot plots and histograms present the number of live and dead cells (%) and the number of GFP+ cells (%) for non-transduced cells (control) and transduced cells (rAAV/DJ) under standard conditions (37 °C) and under hyperthermia (43 °C). The percentage of GFP+ cells was measured in the population of live cells.

The rAAV/DJ vector transduction efficiency in melanoma cells (A375, G-361, and SK-MEL-1) and normal fibroblasts (CCD-1064Sk) under hyperthermia was evaluated (on day 7 of the experiment—see 5.2. paragraph) with the use of a fluorescence microscope, automatic cell counter, flow cytometry, and qPCR. The results are presented in Figure 2, Figure 3, Figure 4 and Figure 5. Hyperthermia enhanced gene transfer in all the tested cell lines. An increase in the number of GFP+ (green fluorescent protein-positive) cells was observed with the following values (automatic counter/FACS): 17%/13% for A375, 7%/8% for G-361, 7%/6% for SK-MEL-1, and 1%/2% for CCD-1064Sk (Figure 3A,B). Figure 4 and Figure 5 show representative dot plots and histograms obtained during the analysis of transduction efficiency via FACS. The percentage of GFP+ cells in the live cell population obtained via FACS is shown in Figure 3B. The percentages of live cells measured with FACS are shown in Figure 3D. A positive effect of hyperthermia on transduction efficiency was also confirmed by the results of qPCR, where the following increases in the vector copy numbers were observed (as compared to transduction at 37 °C): 3.03 × 10^5^ gc (genome copies) for A375, 9.50 × 10^5^ gc for G-361, 11.36 × 10^6^ gc for SK-MEL-1, and 6.86 × 10^6^ gc for CCD-1064Sk (Figure 3C). This is an 85% increase in the number of vector copies for A375, 152% for G-361, 101% for SK-MEL-1, and 75% for CCD-1064Sk, respectively. Of note are the differences between the examined melanoma cell lines occurring both under standard conditions and in response of the cells to hyperthermia. As shown in Figure 3A,B, the most efficiently transducing cell line was A375 (60%/77%), followed by G-361 (20%/27%), and the least efficient was SK-MEL-1 (11%/21%). In normal fibroblasts, the lowest number of GFP+ cells was recorded (4%/5%). The qPCR results additionally emphasized the observed differences between the melanoma cell lines (Figure 3C). Although the A375 line was characterized by the highest number of GFP+ cells, it revealed the lowest number of vector copies (3.57 × 10^5^ gc), while the SK-MEL-1 line with the lowest number of GFP+ cells showed the highest number of vector copies (1.12 × 10^7^ gc). G-361 cells showed 6.23 × 10^5^ gc. Cells of normal fibroblasts were characterized by high values of the vector copies (9.13 × 10^6^ gc), despite having a low GFP+ count. The results of the viability of cells (Figure 3D) showed that the 1 h of hyperthermia had no effect on the number of viable cells. Our aim was to design a protocol that would ensure an increase in gene transfer while maintaining high cell viability. Interesting results were observed for the CCD-1064Sk line, for which an increase in the number of viable cells was determined after exposure to hyperthermia. In contrast, for the A375 line, a 3% decrease in cell viability was observed for cells transduced under hyperthermia. The SK-MEL-1 line, which grew as a suspension, was characterized by a lower viability compared to other cell lines.

### 3.2. Expression of the Receptors for AAV in Melanoma Cells under Standard Conditions and under Hyperthermia

**Figure 6:**
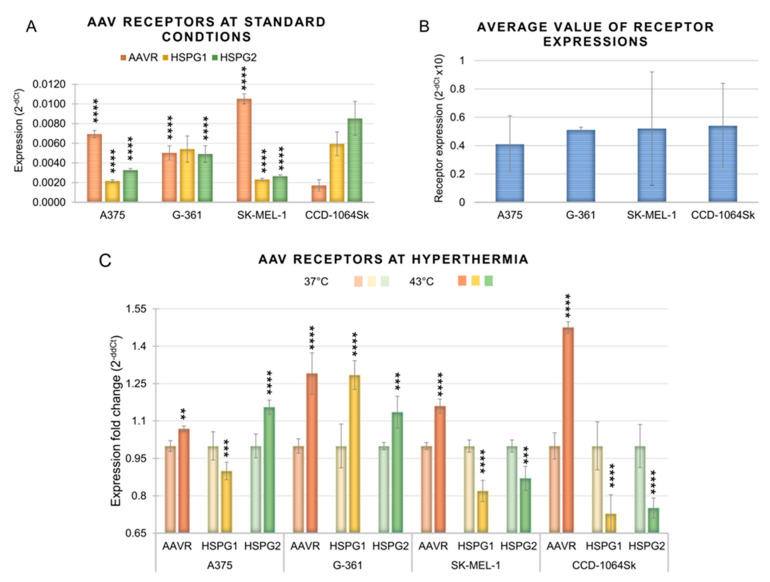
Expression of AAVR, HSPG1, and HSPG2 receptors for AAV in melanoma cells (A375, G-361, and SK-MEL-1) and normal fibroblasts (CCD-1064Sk). The results are presented as a mean of three independent repeats ± SD. (**A**) Expression levels (2^−dCt^) of receptors for AAV under standard conditions—constitutive levels. Statistically significant differences between CCD-1064Sk and cancer cells are marked with asterisks (**** *p* < 0.0001). (**B**) Averaged constitutive expressions (2^−dCt^) of receptors for AAV in the examined cells. The expression levels (2^−dCt^) of the three receptors were averaged for each cell line. (**C**) Changes in the expression (2^−ddCt^) of receptors after exposure to hyperthermia. For each receptor, lighter colors indicate expression at 37 °C and darker at 43 °C. Statistically significant differences between 37 °C and 43 °C are marked with asterisks (** *p* < 0.01; *** *p* < 0.001; **** *p* < 0.0001).

This study evaluated the effect of hyperthermia on the expression of genes encoding the representative receptors for AAV (AAVR, HSPG1, and HSPG2). As shown in Figure 6, melanoma cells and normal fibroblasts differed in their expression profiles of the genes tested both at the constitutive level (under standard conditions) and after exposure to hyperthermia. In the context of searching for the mechanisms of cell transduction stimulation at hyperthermia with the use of rAAV vectors, one should notice that the expressions of receptors for AAV changed in response of the cells to an increased temperature (Figure 6C). All the examined cell lines revealed an increase in the expression of *AAVR*. In the case of the worse transducing SK-MEL-1 and CCD-1064Sk cell lines, only an increase in this receptor was recorded. For the most efficiently transducing A357 cell line, an increase of expression was additionally observed for *HSPG1* after exposure to hyperthermia. G-361 cells responded to hyperthermia with an increase of all receptors. Of note are also differences in the constitutive levels of receptor genes for AAV occurring between the examined lines, which correspond to the determined number of the vector copies (Figure 6A,B). The mean values (Figure 6B) were calculated due to underlying receptor expression differences between the tested cell lines. The average value of three receptor expression levels (2^−dCt^) was calculated for every cell line. For SK-MEL-1 and CCD-1064Sk cells characterized by the highest numbers of rAAV/DJ gene copies, the expression levels of genes encoding proteins involved in AAV transmission to these cells showed higher average levels of 0.52 and 0.54, respectively (Figure 6B).

### 3.3. Expression of HSPs in Melanoma Cells under Standard Conditions and under Hyperthermia

**Figure 7:**
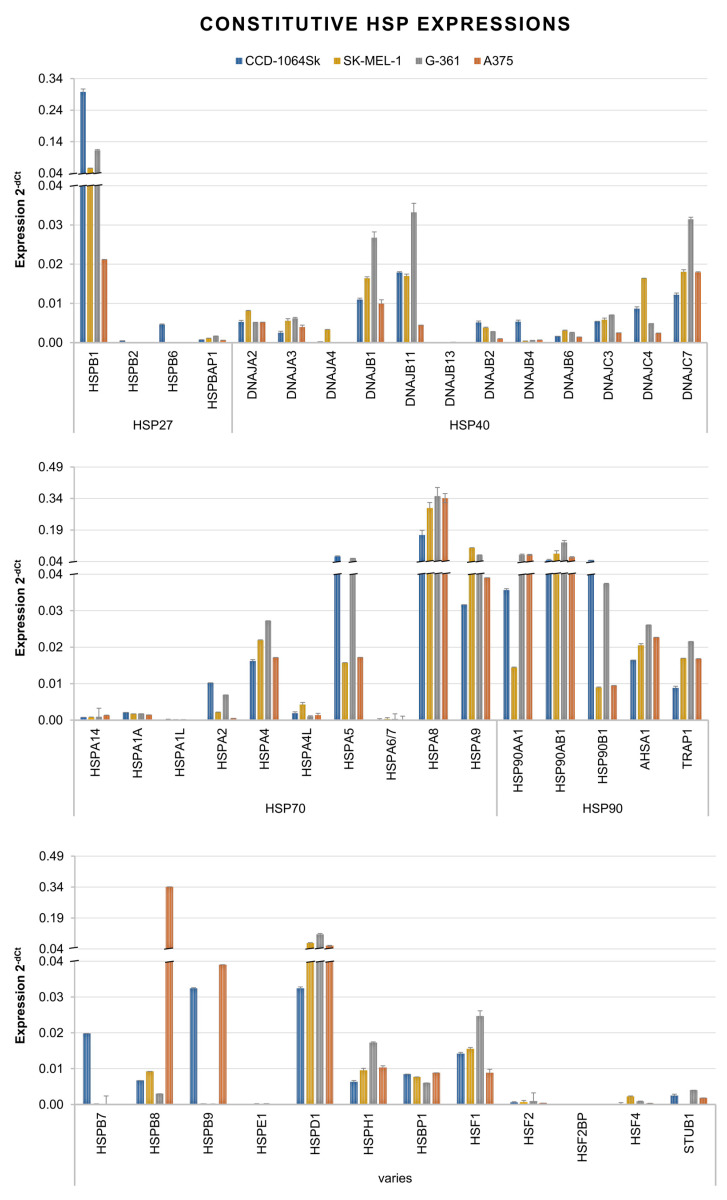
Signatures of the constitutive expression (cells not exposed to hyperthermia) of genes encoding HSPs in A375, G-361, and SK-MEL-1 melanoma cells and normal skin fibroblasts CCD-1064Sk. Results (2^−dCt^) are presented as a mean of two biological repeats ± SD. The analyzed HSPs were grouped into the subfamilies HSP27, HSP40, HSP70, and HSP90, and varies.

**Figure 8:**
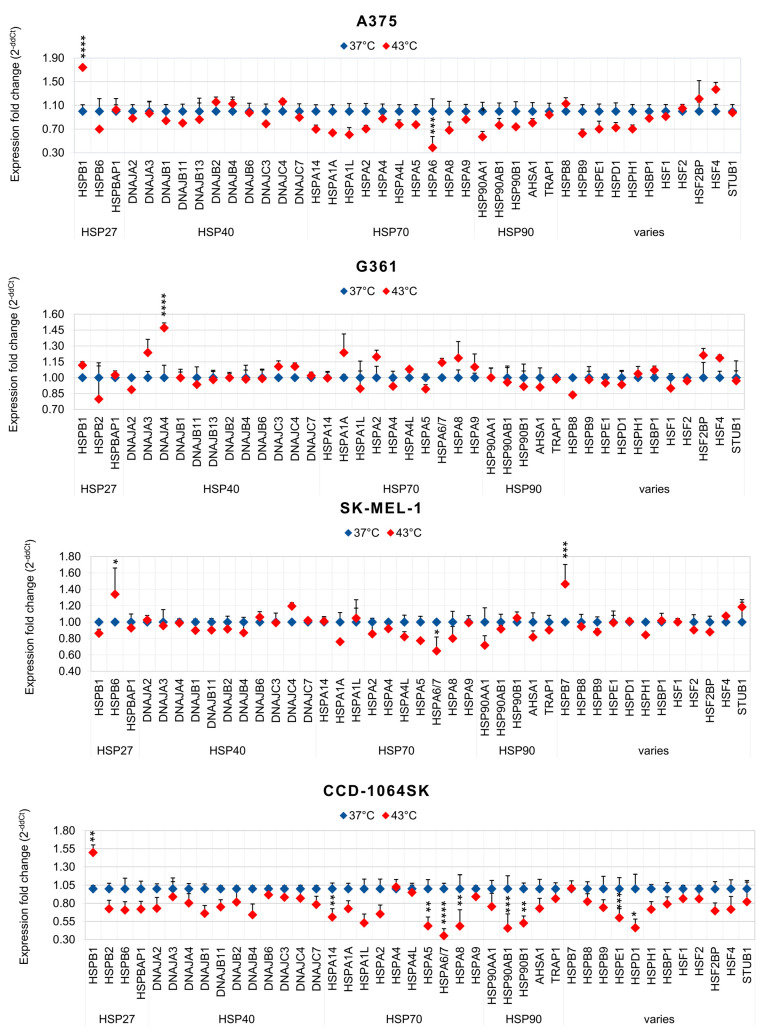
Changes in HSP gene expression in melanoma cells (A375, G-361, and SK-MEL-1) and normal fibroblasts (CCD-1064Sk) exposed to hyperthermia. Results (2^−ddCt^) are presented as a mean of two biological repeats ± SE. Values of expression at 37 °C are marked with blue dots and after exposure to hyperthermia (43 °C) with red dots. Statistically significant differences between 37 °C and 43 °C are marked with asterisks (* *p* < 0.05; ** *p* < 0.01; *** *p* < 0.001; **** *p* < 0.0001).

In search for a response to the mechanism stimulating cell transduction at hyperthermia with rAAV vectors, an analysis of the expression of 44 genes encoding primarily HSPs from the HSP90, HSP70, HSP40, and HSP27 families was performed. Figure 7 presents the profile of HSP gene expression at a constitutive level in the examined cells. Among the genes measured, the highest expression levels (>0.04) were observed for 5–7 HSPs. A375 cells were characterized by a high expression of *HSPA8 (HSP70)*, *HSP90AA1*, *HSP90AB1 (HSP90)*, *HSPB8 (HSP22)*, and *HSPD1 (HSP60)*. G-361 cells were characterized by *HSPB1 (HSP27)*, *HSPA5*, *HSPA8*, *HSPA9 (HSP70)*, *HSP90AA1*, *HSP90AB1*, and *HSPD1.* For SK-MEL-1 cells, the highest expression levels were measured in the case of *HSPB1*, *HSPA8*, *HSPA9*, *HSP90AB1*, and *HSPD1*, and normal fibroblasts CCD-1064Sk were characterized by *HSPB1*, *HSPA5*, *HSPA8*, *HSP90AB1*, and *HSP90B1 (HSP90)*. It can be observed that all the examined cells were characterized by an expression > 0.04 for *HSP90AB1* and *HSPA8*, and melanoma cells additionally showed an increased expression of *HSPD1*. Cell exposure to hyperthermia caused changes in HSP expression; the results are shown in Figure 8. For the most efficiently transducing A357 cells, an increase of expression was observed for *HSPB1*, *DBAJB2*, *DNAJB4*, *DNAJC4 (HSP40)*, *HSPB8*, *HSF2BP*, and *HSF4*. In the case of G-361 cells, an increase in expression was recorded for *HSPB1*, *DNAJA3*, *DNAJA4*, *DNAJC3*, *DNAJC4 (HSP40)*, *HSPA1A*, *HSPA2*, *HSPA4L*, *HSPA6*, *HSPA7*, *HSPA8*, *HSPA9 (HSP70)*, *HSPH1 (HSP110)*, *HSBP1 (HSF)*, *HSF2BP*, and *HSF4*. SK-MEL-1 cells only responded with an increased expression of *HSPB6 (HSP27)*, *DNAJC4*, *HSPB7 (HSP7)*, and *STUB1*. The least transducing CCD-1064Sk cells were characterized by an increase in one gene, *HSPB1*. These results show that all the examined cells revealed an increase in the expression of one gene of the HSP27 family (*HSPB1* and *HSPB6*); additionally, melanoma cells were characterized by an increase in *DNAJC4* (*HSP40*), which may indicate a significant role of these proteins in stimulating transduction under hyperthermia. It is worth noting that despite the similarities between the examined cell lines, the melanoma cells are a molecularly heterogeneous group, which may explain a varied response to hyperthermia.

## 4. Discussion

Gene therapy, with the whole range of molecular biology tools, perfectly fits in the current needs of medicine with respect to the treatment of patients with melanoma. One of the major challenges of gene therapy is the selection of an adequate gene carrier which will ensure a high gene transfer since it decides on the treatment efficacy. For this reason, methods ensuring a high gene transfer efficiency are searched for [47]. Ongoing studies have shown that hyperthermia enables improvement in the transduction efficiency with the use of rAAV [27,28,29,30]. A growing number of clinical trials concerning gene therapy with these vectors (n = 350; 2023), and the registration of subsequent medicinal products based on these rAAV constructs, have shown their importance in the field of medicine [34,48].

This research points to the possibility of improving melanoma cell transduction efficiency with rAAV/DJ vectors by means of hyperthermia. In our previous works, we demonstrated that the stimulation of transduction using an increased temperature is possible both in cells of ovarian cancer and colorectal cancer [27,28]. In the present study, the mosaic vector rAAV/DJ, under the control of a strong CAG promoter, was used for the transduction of melanoma cells (A375, G-361, and SK-MEL-1) and normal skin CCD-1064Sk fibroblasts. Hyperthermia (1 h; 43 °C) enhanced transduction efficiency in all the examined cells. The cells differed in terms of the number of GFP+ cells measured and the number of vector gene copies. As shown in Figure 3, the most efficiently transducing cell line at hyperthermia was A375 (87% GFP+, 6.59 × 10^5^ gc), followed by G-361 (50% GFP+, 1.57 × 10^6^ gc), and the least efficient was SK-MEL-1 (21% GFP+, 2.26 × 10^7^ gc). The skin fibroblasts CCD-1064Sk revealed the lowest number of GFP+ cells (7% GFP+, 1.60 × 10^7^ gc). Interestingly, the examined cells were characterized by a negative correlation between the number of GFP+ cells and the number of vector copies, which was observed both under standard conditions and under a higher temperature. Cells exhibiting a high level of transcriptional activity (high GFP+ expression with a low number of vector copies) respond more effectively to hyperthermia, which was observed in the A375 cells. At the same time, the cells with the highest number of vector copies under standard conditions reached their highest increases in the vector copy number after exposure to hyperthermia (SK-MEL-1 and CCD-1064Sk). A similar correlation was observed in our previous study on colorectal cancer cells (RKO, HT-29, and LS411N) [27], where the cells best responding to hyperthermia—RKO (with the highest number of GFP+ cells; 51%)—revealed the lowest number of vector copies, while the cells with the highest number of vector copies—HT-29—showed their highest increment (by 1.62 × 10^6^ gc, 152%) [27]. The effect of hyperthermia of transduction efficiency was also evaluated by other authors. A study by Zhong L. et al. [29] demonstrated that exposure of HeLa cells to 42.5 °C for 4 h caused a 6-fold increase in rAAV vector transduction efficiency as compared to standard conditions. In the present study, a shorter time of exposure to hyperthermia (1 h) was selected, since the hyperthermia procedure in clinics lasts predominantly from 30 min to 90 min [13,15]. Attempts have also been made to use hyperthermia to improve cell transduction efficiency with the use of other viral vectors. In their study, Wang J. et al. [49] used hyperthermia with radiofrequency to reinforce the therapeutic effect of the construct based on the system of herpes simplex virus and thymidine kinase/ganciclovir (HSV-TK/GCV) in the treatment of hepatocellular carcinoma. These authors demonstrated that the procedure they used enhances the anti-cancer activity of HSV-TK/GCV [49].

In works describing the mechanisms of gene transfer with the use of AAV, interest has been aroused by receptors for AAV which significantly affect the transduction and tropism of these vectors [50,51,52]. A special importance has been attached to the recently discovered AAVR receptor KIAA0319L. It has been shown that AAVR has a central role in binding AAV to cells and is essential for the internalization and efficient transduction of a wide range of cells [50]. An important role in AAV infection is also assigned to HSPGs, which take part in the initial interaction of the virus with the cell [52]. Therefore, the present study evaluated the expression of AAVR, HSPG1, and HSPG2. As shown in Figure 6, the examined cell lines differed in the expression levels of the examined genes. The results correspond to data reported in the literature, indicating that there are rAAV transmission profiles specific for different cells [53], and that there are differences in these receptor’s expression levels between cell lines [50]. The used cell lines have the same tumor origin, but differ in their etiology, morphology, and genomic mutation [45], which may be the reason for the variability of AAV receptor expression between them. The results obtained in the present study showed that hyperthermia induced changes in the expression of the examined genes (Figure 6C). All the analyzed cells revealed an increased AAVR expression levels, and the cells transducing at a higher efficiency additionally showed an increase in the other genes HSPG1 (A375 and G-361) and HSPG2 (G-361). In our previous papers, we observed similar changes in the expression of genes encoding receptors for AAV occurring as a result of hyperthermia, which corresponded with cell transduction efficiency [27,28]. The obtained results suggest that one of the mechanisms leading to improvements in transduction efficiency by means of hyperthermia is an increase in the expression of receptors for AAV (especially AAVR), which is associated with an increased number of rAAV/DJ vector copies in cells.

In the present study, we assessed a panel of HSPs in order to better understand the changes occurring in melanoma cells due to hyperthermia, and to indicate which HSPs could play a role in stimulating the transduction of rAAV/DJ at an increased temperature. In the context of viral infections, including the COVID-19 pandemic, importance is attached to the participation of HSPs in the virus life cycle [23,24,25]. HSPs are important for the expression of viral genes at the transcriptional and translational levels. HSPs facilitate the entry into and release of viruses from cells [23,24,25]. Studies by Zhong L. et al. [29] and Zhao W. et al. [30] showed an effect of HSPs on the transduction of HeLa cells with rAAV vectors. These authors demonstrated that the improvement in transduction efficiency is caused by the HSP90 and FKBP52 complex (proteins of the immunophilin family). The exposure of cells to an increased temperature causes the dephosphorylation of FKBP52 and the dissociation of the FKBP52-HSP90 complex, leading to an increase in gene transfer efficiency. An isolated enhancement of HSP90 expression leads to the stabilization of the FKBP52-HSP90 complex, a suppression of second-strand DNA synthesis, and a decrease in transduction efficiency, which indicates the participation of other proteins in the stimulation of gene transfer via rAAV [29,30]. In the present study, the analysis of HSP signatures of the analyzed cell lines both under standard conditions (Figure 7) and after exposure to hyperthermia (Figure 8) revealed that certain HSPs are characterized by a high level or an increased expression in all melanoma cells. Under standard conditions (37 °C), melanoma cells were characterized by a high level (>0.04) of *HSPD1* (HSP60), *HSPA8* (HSP70), and *HSP90AB1* (HSP90), and a medium level (0.02–0.04) of *HSPB1* (HSP27), *HSPA9* (HSP70), and *AHSA1* (HSP90). A specific profile of genes encoding HSPs at the constitutive level may be an indicator of potential cells whose exposure to hyperthermia will improve gene transfer. The analyzed melanoma cells were characterized by an increased expression of the HSP90 genes, which may be one of the causes of increased transduction efficiency, but also, as reported by data in the literature, may indicate an advanced stage of carcinogenesis [54]. HSPs play an important role in the regulation of apoptosis: HSP10 and HSP60 are pro-apoptotic, while HSP27, HSP70, and HSP90 are anti-apoptotic [55]. The characteristic HSP profile in our work, with increased expression levels of mostly anti-apoptotic HSPs, may be one of the reasons of observed high viability of adherent cells under standard conditions, as well as after exposure to hyperthermia (Figure 3D). Of note is the fact that despite similarities in HSP signatures between the analyzed cell lines, the cells showed varied levels of expression of specific genes, which may have a direct influence on the transduction efficiency at hyperthermia. The exposure of cells to hyperthermia caused changes in the expression levels of the analyzed HSPs, and the analyzed cells were characterized by different HSP signatures. As shown in Figure 8, for the most efficiently transducing cell line A375, an increased expression was observed in *HSPB1* (HSP27), *DNAJB2*, *DNAJB4*, *DNAJC4* (HSP40), *HSPB9* (HSP70), *HSF2B4*, and *HSF4* (heat shock factors). The indicated specific HSP signature may stimulate rAAV transduction. Interestingly, an increase in HSP27 expression was observed after exposure to a higher temperature in all melanoma cells: *HSPB1* (A375 and G-361), and *HSPB6* (SK-MEL-1) and HSP40: *DNAJC4* (A375, G-361, and SK-MEL-1). Comparing the results with our previous studies, it was observed that *HSPB1* was characterized by an increased expression after exposure to hyperthermia in seven of ten analyzed cell lines (colorectal cancer, ovarian cancer, and melanoma), whereas increased *DNAJC4* expression was only observed in five of ten cell lines (colorectal cancer and melanoma), which may indicate their important role in the process of stimulating rAAV/DJ transduction at hyperthermia. The above observations confirm scientific reports on the special role of HSP27 in viral infections [56,57,58]. The knockout of *HSP27* causes suppression of enterovirus A71 replication [56]. *HSP27* expression rapidly increases after coronavirus infection (4 h), which suggests its significant role in early replication. During infection with porcine circovirus type 2, containing single-stranded DNA, there is an increase in the expression of HSP27 (in a phosphorylated form). Suppression of HSP27 expression leads to the stoppage of virus replication [58]. As for HSP40, scientific evidence suggests that this protein regulates the initiation of viral DNA and RNA replication by modulating the activity of polymerase, the replication complex, and nuclear transport [23].

What is worth noting is the participation of HSPs in the regulation of the structure of the cell membrane. HSPs interact with lipids and may also enhance endocytosis. It has been indicated that sHSP, HSP60, HSP70, and HSP90 may participate in these processes [59,60,61]. Interestingly, HSP70 supports clathrin-dependent endocytosis [62], which is known to be one of the ways that rAAV enters cells [63]. In the work of Vega V. et al. [64], it was shown that the uptake of macromolecules increased in cells in response to heat shock. The authors showed that HSP70 was responsible for the enhancement of endocytosis. In our work, cells were characterized by a high expression (>0.04) of genes encoding HSPB1 (HSP27), HSPD1 (HSP60), HSPA5, HSPA8 (HSP70), HSP90AA1, and HSP90B1 (HSP90) (Figure 7), which, as shown in other works, are located on the surface of cells [59]. One of the mechanisms responsible for the increase in the efficiency of cell transduction with rAAV vectors under hyperthermia may be the participation of HSPs in changing the permeability of cell membranes.

## 5. Conclusions

In conclusion, the obtained results show that rAAV/DJ vector transduction efficiency in melanoma cells increases at an elevated temperature. What is more, transduction stimulation at hyperthermia is associated with signatures of HSPs and receptors for AAV of the analyzed cells, which change after exposure to an elevated temperature. A key role in the improvement of gene transfer may be played by *AAVR*, *HSPB1*, *HSP6*, *DNAJC4*, *HSPD1*, *HSPA8*, *HSPA9*, *HSP90AB1*, and *AHSA1*. This study, as well as our previous studies [27,28], provides arguments for the use of hyperthermia as a factor enabling the stimulation of cell transduction with rAAV vectors, thereby providing tools for the improvement in the efficacy of gene therapy based on these gene carriers. The present paper provides information which may be useful in designing clinical protocols of gene therapy using hyperthermia in patients with melanoma.

## Data Availability

All data reported in this paper are contained within the manuscript.
